# Probing Antimicrobial Halloysite/Biopolymer Composites with Electron Microscopy: Advantages and Limitations

**DOI:** 10.3390/polym13203510

**Published:** 2021-10-13

**Authors:** Kirill Cherednichenko, Dmitry Kopitsyn, Svetlana Batasheva, Rawil Fakhrullin

**Affiliations:** 1 Department of Physical and Colloid Chemistry, Faculty of Chemical and Environmental Engineering, National University of Oil and Gas «Gubkin University», 65 Leninsky Prospekt, 119991 Moscow, Russia; cherednichenko.k@gubkin.ru (K.C.); kopicin.d@inbox.ru (D.K.); 2 Institute of Fundamental Medicine and Biology, Kazan Federal University, Kreml uramı, 18, 420008 Kazan, Republic of Tatarstan, Russia; svbatasheva@gmail.com

**Keywords:** halloysite nanotubes, biopolymer, clay/polymer composites, electron microscopy, biofilms, antimicrobials

## Abstract

Halloysite is a tubular clay nanomaterial of the kaolin group with a characteristic feature of oppositely charged outer and inner surfaces, allowing its selective spatial modification. The natural origin and specific properties of halloysite make it a potent material for inclusion in biopolymer composites with polysaccharides, nucleic acids and proteins. The applications of halloysite/biopolymer composites range from drug delivery and tissue engineering to food packaging and the creation of stable enzyme-based catalysts. Another important application field for the halloysite complexes with biopolymers is surface coatings resistant to formation of microbial biofilms (elaborated communities of various microorganisms attached to biotic or abiotic surfaces and embedded in an extracellular polymeric matrix). Within biofilms, the microorganisms are protected from the action of antibiotics, engendering the problem of hard-to-treat recurrent infectious diseases. The clay/biopolymer composites can be characterized by a number of methods, including dynamic light scattering, thermo gravimetric analysis, Fourier-transform infrared spectroscopy as well as a range of microscopic techniques. However, most of the above methods provide general information about a bulk sample. In contrast, the combination of electron microscopy with energy-dispersive X-ray spectroscopy allows assessment of the appearance and composition of biopolymeric coatings on individual nanotubes or the distribution of the nanotubes in biopolymeric matrices. In this review, recent contributions of electron microscopy to the studies of halloysite/biopolymer composites are reviewed along with the challenges and perspectives in the field.

## 1. Introduction

Biofilm is the predominant mode of life for a majority of microorganisms [1]. It is a complex composition of various microorganisms/bacteria attached to a biotic or abiotic surface and embedded within an extracellular polymeric matrix produced by constituent microorganisms [2,3]. This matrix is typically a conglomeration of extracellular polysaccharides, proteins, lipids and DNA [2,3,4] and may account for more than 90% of the biofilm dry mass [5].

Microbes assembled in biofilms are behind numerous positive and negative impacts on our everyday life. On the one hand, biofilms are employed for wastewater treatment at sewage treatment plants. The biofilms attached to water filters extract and digest organic compounds, suspended solids, pathogens and microorganisms [6,7,8]. They can also be applied for electrical energy generation/storage by recycling biomass or complex organic wastes in microbial fuel cells [9,10,11,12]. On the other hand, a majority of human bacterial infections are caused by biofilms. For instance, 60–70% of hospital-acquired infections are caused by biofilm contamination of the medical device surfaces [5,13]. The formation of a biofilm is usually a precedent step to marine biofouling [14,15,16,17] leading to ships slowdown, and, hence, extra fuel consumption. Moreover, the microorganisms in biofilms can catalyse chemical and biological reactions leading to surface degradation, e.g., corrosion of underwater constructions [18] or dental caries [1]. 

The major issue related to biofilms is their almost irreversible attachment to the colonized surface. The biopolymeric matrix produced by microorganisms plays a significant role in biofilm resistance to various stresses and harsh environments as well as enhances the ability of cells to adhere to the surface [19]. For instance, extracellular DNA contained in the matrix can bind to antimicrobial agents, limit their diffusion and, thus, significantly reduce their action against microbial cells in the biofilm [1,5,20,21,22,23]. The limited oxygen content, acidic pH and slow metabolism of cells within the biofilm may considerably influence the efficacy of some antibiotics [1,24]. Resistance of biofilm cells to antibiotics can be even higher than that of single cells. In biofilms, the individual cells may undergo mutations that also contribute to the enhanced antibiotic resistance [24].

Since the first report on biofilms in 1943 [25] the strategies to prevent/retard biofilm formation/development in various conditions have been continuously developed. Although a number of different strategies have been elaborated for the particular surface type, environments, biofilm type and its stage of development, all of them can be divided into two main groups: physical and chemical methods. The first group includes mechanical treatment (e.g., grinding, brushing, lapping, and polishing) [1,5,16], ultrasonic treatment [5,26], non-thermal plasma [1,5,27,28], UV light and gamma radiation [27] of the colonized surfaces. Since the killing of biofilm living cells by the antimicrobial agents is significantly impeded by the extracellular polymeric matrix, the physical methods become an essential preparatory step to biofilm removal [1,5,16]. However, even though physical methods can help to remove a considerable part of microorganisms, they cannot ensure the complete removal of microbes from the surface due to some surface imperfections like cracks, crevices, gaskets, etc., which become new centres of biofilm growth. Hence, the physical methods should be combined with chemical methods implying surface disinfection using various biocides (e.g., hydrogen peroxide, quaternary ammonium compounds, isothiazolones, organosulfur compounds, peracetic acid, NaClO, ozone, etc.) [5,16,26,27,28,29,30]. Electrochemical treatment [5,16,31] is another example of the successful combination of the physical and chemical methods. The water electrolysis and the generation of strong biocides/oxidants such as Cl_2_, HOCl, NaOCl, H_2_O_2_ or hydroxyl free radicals can significantly eradicate biofilm components. On the other hand, since the majority of bacterial cells are usually negatively charged, the negative polarization of the surface can lead to a repulsive electrostatic interaction with bacterial cells and, thus, weak cell attachment [32,33,34,35,36]. Summing up, the combination of physical and chemical methods demonstrates effective biofilm eradication, however all the protocols mentioned above are applicable for the cases when biofilm is already formed.

The prevention of biofilm attachment and growth requires more sophisticated approaches. Indeed, the surface properties play a great role at the stage of the initial attachment of bacteria and microorganisms and further biofilm development. The interaction between biofilm cells and the surface can be regulated through the change in the surface hydrophobicity [5,16,26,30], media pH [26], roughness [1,5,16,27,30,37], surface charge [30], and chemical composition (e.g., in case of metal surfaces: type of metal, metal oxidation state, its reactivity) [1,16,19,27]. Since the attachment in general will occur most readily on rougher and hydrophobic surfaces [16,30], the proper modification of surface roughness/topography and use of various surfactants [16,26,30] can significantly inhibit biofilm formation even without the use of any antimicrobial agents [16,27]. The modification of the surface by metal ions (e.g., Cr, Co, Cu, As, Hg, Pb, Sn, Cd, etc.) in the form of an alloy additive [17], a part of biocide coating [30] or as nanoparticles grafted to the surface [1,24] can lead to disruption of many crucial biofilm cells processes [1,16,37,38,39]. The main concern about the use of ‘toxic’ metals or other inorganic species for biofilm inhibition is the large dosage needed and, consequently, their adverse environmental impact. For instance, due to toxicity considerations, they cannot be used for the purification of water intended for human use.

In this regard “biocontrol” or “green strategy” involving the use of polysaccharides [1,40], enzymes [7,26,27,28,29,30], (poly)peptides [24,26], proteins [41,42,43,44], liposomes, bacteriophages and bacteria [1,26,27] to control biofilm formation seems to be more beneficial. For instance, chitosan (the second most abundant polysaccharide in nature) was found to be an effective antimicrobial agent [40]. One of the polysaccharide antibiofilm activity mechanisms is interference to cell-to-cell and cell-to-surface bacteria interactions [45]. 

Enzymes demonstrated high activity against different types of biofilm by interacting with DNA, lipids and carbohydrates of biofilm extracellular matrix and thus breaking the intercellular links and causing biofilm dispersal to individual bacteria. Production of such enzymes is one of the features of bacteriophages (viruses that can infect microorganisms constituting a biofilm) [1]. As soon as biofilm integrity has deteriorated the application of antibiotics/drugs [24], biocides and surfactants [26] or even physical treatments [30] becomes more effective. 

Peptides (e.g., bacteriocins) can also target different components of biofilm cells and inhibit biofilm development [24,26,46]. These biopolymers possess enhanced physicochemical properties like ameliorated diffusion properties and stability in different pH conditions. Sometimes there is no need to isolate the peptides from living organisms, as bacteriocins (as well as biosurfactants with anti-adhesive properties) may be produced by some bacteria [24,26]. Moreover, the antibiofilm activity of the particular bacteria involves modification of physical and chemical properties of the colonized surface as well as exclusion/competition phenomena within a biofilm, hence, leading to significant interference with biofilm adhesion and maturation [1]. Summing up, the biopolymers and other antimicrobial bioagents show highly specific inhibition and a broad spectrum of activity, non-toxicity towards human and animal tissues, blending compatibility with organic matrixes such as textile, paints, polymer, etc. However, their relatively low stability (e.g., low decomposition temperature and short life expectancy) cannot be ignored [40].

Significant efforts were made to overcome some disadvantages of synthetic and natural antimicrobial agents, enhance their penetration through the biofilm polymer matrix and prolong their antimicrobial activity. Ideally, the antimicrobial agents should be released/activated at a particular moment when their eradication effect would be maximal. Such requirements can be fulfilled by antimicrobial composites: they can enhance antibiotics delivery by overcoming extracellular polymeric matrix barrier, provide firm fixation of an antimicrobial agent on the surface and controlled biocide release for a certain time period or release triggered by a change in conditions, e.g., pH. They can also change the surface topography, hence, its roughness and hydrophobicity. Composite materials must be non-toxic and environmentally safe, i.e., be chemically inert while resistant to the harsh environments. Finally, yet importantly, the composites should be cheap to be attractive from the economical point of view. Over the last few decades, a great variety of materials has been tested as a potential support for antimicrobial agents in such fields as antifouling coatings [47,48,49,50,51,52] or medicine [53,54,55,56,57,58,59,60]. Nowadays the natural carriers (e.g., biofibres, biofilms, natural nanocontainers) are of great popularity caused by growing environmental issues [58,61,62,63]. The clays (natural aluminosilicate ceramic materials) are considered as perspective carriers/support for synthetic and organic antimicrobial agents owing to their outstanding properties, as well as abundance and, hence, the low price [40,53,64,65,66,67,68,69,70,71,72,73,74,75,76,77].

## 2. Halloysite as a Perspective Carrier/Support for Biopolymers

**Figure 1:**
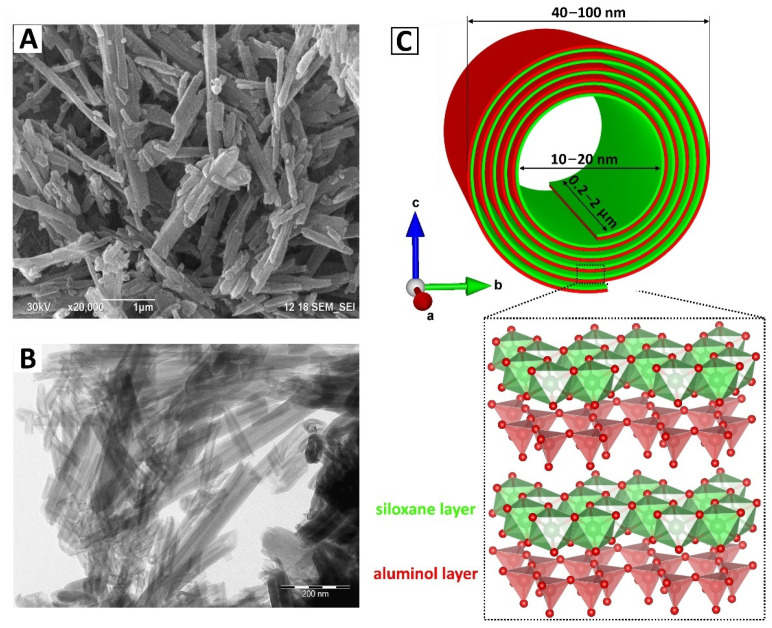
Morphology (visualized by transmission electron microscopy (TEM) (**A**) and scanning electron microscopy (SEM) (**B**)), and schematic representation of crystal structure (**C**) of halloysite.

Halloysite nanotubes (HNTs), natural aluminosilicate nanotubes, are a clay mineral of the kaolin group. It is outstanding within the clay family thanks to its unique tubular structure formed under favourable geological conditions [78]. It is a rather abundant mineral and, hence, of low cost owing to large natural deposits [79,80,81,82]. The aluminosilicate nanosheets are rolled in nanotubes with length from 0.2 to 2 μm, external diameter from 40 to 100 nm, and internal diameter from 10 to 20 nm. The space between nanosheets usually contains water molecules, hence, the halloysite chemical formula is Al_2_(OH)_4_Si_2_O_5_·*n*H_2_O, where *n* = 4 for raw mineral, while heating and/or vacuum treatment can remove water. Depending on water content the distance between neighbour nanosheets can shrink from 1 nm (*n* = 4) to 0.70 nm (*n* = 2) [73,79,80]. Scanning and transmission electron microscopy and schematic representation of HNT are shown in Figure 1.

**Figure 2:**
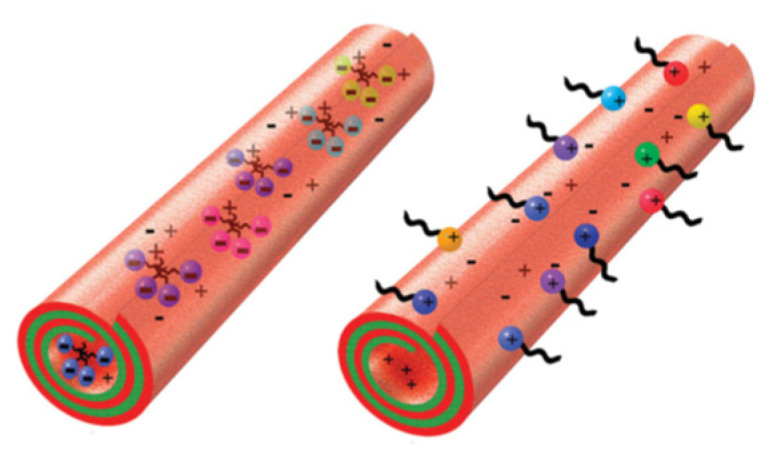
Scheme of selective anionic and cationic amphiphilic molecules adsorbed on the inside and outside of halloysite nanotubes. Reproduced with permission from Ref. [79]. Copyright 2016 John Wiley and Sons.

The inner and outer halloysite nanotube surfaces consist of corner-shared tetrahedral SiO_4_ layer and edge-shared octahedral AlO_6_ layer with an internal Al–OH groups respectively [79,80]. Such a unique surface chemistry leads to different surfaces charges of halloysite: the inner lumen has a strong positive charge, while outer surface has a weak negative charge [79,80]. This fact allows the selective absorption/loading of charged compounds on the either nanotube surface (Figure 2). Moreover, higher hydrophobicity of HNT outer surface can be achieved by appropriate modifications, which results in firm retention of hydrophilic compounds inside the lumen [73].

Natural halloysite exhibits comparatively low zeta-potential (approximately −30 mV). However, modification of the internal surface of the tubes or loading of negatively charged substances can drastically increase the zeta-potential to −50 to 60 mV making possible the formation of stable water-based dispersions applicable for convenient antibacterial sprays [53]. The mechanical tests of HNTs revealed high Young’s modulus (130 GPa) which decreases as outer diameter increases, and surprising flexibility (the nanotube can be bent up to nearly 90° without fracturing) [80]. The measured density and porosity values of HNT are 2.53 g/cm^3^, 50–60 cm^2^/g, respectively [81]. Thanks to its outstanding mechanical properties halloysite seems to be the best option for supporting/encapsulation of biopolymers and organic antimicrobial agents whose application is limited by low stability and endurance [40]. In addition, HNT has high biocompatibility and very low toxicity [53,73,80]. Thus, because of the different surface charges, high surface area and aspect ratio, good porosity and capacity, ability to firmly retain loaded compounds and long-lasting release, HNTs are attractive carriers of drugs or antibiotic agents and a support for antimicrobial coatings [53,64,65,66,67,68,69,73,74,75,76,77,78,79,80,81,82,83,84,85,86,87,88,89,90,91,92,93,94,95,96,97].

## 3. Synthesis of Biopolymer/Halloysite Nanotube (HNT) Composites

The HNT-based hybrid materials may be obtained either by grafting or loading of compounds in the lumen as well as their adsorption on the external surface or by the combination of both methods [53]. The grafting implies chemical modification of halloysite internal or external surfaces through the chemical reaction of hydroxyl groups with various organic compounds [98,99,100,101,102,103,104,105]. The interlayer surface can also be modified through grafting [104]. However, this modification is limited by the steric factors since guest molecules should be small enough to intercalate. A compound loading/absorption into/onto halloysite can be performed by stirring nanotubes in compound solution usually assisted with sonication and vacuuming or freeze drying [53,69,79,80,81,82,91,99,100,101,102,103,104,105,106]. The efficiency of loading/absorption and long-lasting release significantly depend on the compound nature and type of interactions involved (ion exchange, hydrogen bonding and van der Waals forces). For instance, halloysite modification with surfactants is based on the preferential adsorption of the polar groups of the surfactant onto the high-energy surface of halloysite by electrostatic interaction [104]. The loading of high-molecular-weight biomacromolecules usually requires lumen modification to enhance loading capacity. However, sometimes simple milling of dry components can result in the effective “wrapping” of HNTs by DNA [86]. Recently freezing of water solution was employed for halloysite loading with Au nanoparticles [107]. Since this approach proved its loading efficiency there are strong grounds to believe that it will be further successfully implemented for other compounds/drugs.

A good example of combination of synthetic approaches was demonstrated in [99] for obtaining HNTs functionalized by chitosan for delivery of curcumin. First, as a result of the consecutive reaction of the external hydroxyl groups with 3-aminopropyltriethoxy silane (APTES), succinic anhydride and chitosan, the latter was successfully grafted to the halloysite surface. Then curcumin was loaded by stirring and ultrasonic treatment.

Evidently, the potency of as-synthesized drug carriers or antimicrobial HNT-based composites considerably depends on the quality of biopolymer loading/absorption. In this regard, the importance of an adequate and informative analysis of the obtained materials is hard to overestimate. In the last few decades, a number of analytical techniques have been employed for the comprehensive analysis of biopolymer/HNT composites.

## 4. Analysis of Biopolymer/HNT Composites

The differential thermal analysis (DTA), thermogravimetric analysis (TGA), X-ray diffraction (XRD), X-ray photoelectron spectroscopy (XPS), Fourier-transform infrared spectroscopy (FTIR), UV-vis-near IR spectroscopy, confocal microscopy and electron microscopy are popular analytical methods for biopolymer/HNT composites characterization.

Optical microscopy is one of the easiest and straightforward ways to investigate microstructure of composites formed by halloysite with different biopolymers. It can be applied for visualization of continuous composite films or discrete composite microparticles, such as emulsions formed by HNTs with hydrophilic or hydrophobic particle surfaces [108,109]. The wettability of halloysite nanotubes was tuned by surface modification with chitosan biopolymer and subsequent pyrolysis in an inert atmosphere. The size of emulsion droplets depending on the level of surface carbonization was analysed with the help of optical microscopy [108]. In another case, optical microscopy was used to demonstrate increased microorganisms adhesion to the surface of oil droplets in an emulsion formed by hydrophobized HNTs [109]. However, this analytical approach is limited by relatively low resolution while the interpretation of the obtained results is rather doubtful for the samples with complex morphology. For imaging the distribution of halloysite in *C. elegance* worms enhanced dark-field microscopy was applied [110]. Laser scanning confocal microscopy or fluorescent microscopy are suitable for such specific tasks as visualization of biopolymer/HNT composites with fluorescent properties or with special fluorescent labelling [82,93,101].

Apart from microscopy, the analysis of the size of discrete biopolymer/HNT composites in the form of particle suspension can be performed with the help of the dynamic light scattering (DLS) method. It should be underlined that DLS is intended for size evaluation of spherical particles, whereas in the case of long cylinders with a high aspect ratio such as HNTs it gives not real but apparent sizes. In spite of the fact that this parameter can be sometimes used as a reference value for indirect confirmation of HNT surface modification with a biopolymer [102,111], it is still not reliable due to the small change in size after halloysite surface modification that is usually of a few nanometers. In contrast to DLS, a change in zeta potential is a more reliable confirmation of surface modification of functionalized halloysite. This value can be significantly increased after surface modification or turned from negative for pristine HNTs to positive for biopolymer/HNTs composites [100,102]. Zeta-potential is of high importance for characterization of discrete biopolymer/HNT composites due to its correlation with suspension stability and possibility of formation of ordered structures. For example, pristine HNTs form highly ordered patterns («coffee-ring» deposits) on the edge of droplets after evaporation [112], meanwhile HNTs modified with sodium polystyrene sulfonate form more scalable patterns due to an increase in surface charges of the tubes and improved dispersion stability [113].

Other methods for confirmation of successful modification of halloysite surface with biopolymers is the analysis of hydrophobic/hydrophilic properties. The hydrophobicity of different chitosan grafted HNTs was verified by extraction of modified halloysite with toluene/water mixture [100,114]. Pristine HNTs with hydroxyl groups on the surface and HNTs modified with carboxylic groups are hydrophilic and therefore located in the bottom (water) layer of the toluene/water mixture while HNTs modified with amino groups and chitosan-grafted HNTs exhibit hydrophobic properties and are located in the toluene layer. A quantitative analysis of the nanoparticles distributed between the oil and water phases was performed using the gravimetric method [100,115]. Another approach for quantitative analysis of hydrophobic/hydrophilic properties of biopolymer-modified halloysite is the contact angle measurement after water deposition onto the substrate prepared with modified halloysite [100].

The spectroscopy techniques are widely applied for the analysis of halloysite-based composites. For instance, the kinetics of drug release can be analysed with UV-Vis spectroscopy by measuring target compound contents in solution after its loading or release [97,100,116]. To evaluate the phase formation within composite specimens Fourier-transform infrared (FTIR) spectroscopy in a wavenumber range of 400–4000 cm^−1^ was successfully applied [96,97,99,100,101,102,106,117]. Characteristic vibration bands in IR spectra confirm successful functionalization of the halloysite surfaces with a target component. Comparison of vibration bands in halloysite before and after adsorption can be used for obtaining information about packing density of organic moieties at the interface [92].

X-ray diffraction (XRD) may be useful in studies of various biopolymer matrices, containing halloysite [96]. Since XRD is sensitive to crystalline substances, the presence of the halloysite diffraction peaks can be evidence of successful dispersion of HNTs in the biopolymer matrix which is usually amorphous. Furthermore, the accurate analysis of halloysite reflections (e.g., (001) plane diffraction peak) can suggest the shielding effect on the layer spacing of HNTs by biopolymer macromolecules [100,102].

Another way to confirm the presence of a new substance on the halloysite surface is X-ray photoelectron spectroscopy (XPS) analysis. In [102], high-resolution XPS spectra of nitrogen (N1s) and carbon (C1s) were used for detection of polyamidoamine on halloysite nanotubes. Determination was based on the appearance of new peaks in the composite spectra compared to the spectra of pristine halloysite. XPS can also indicate whether both surfaces of the nanotube were modified. Thus, the difference between the high-resolution XPS spectra of aluminium and silicon in HNT and HNT-loaded gellan gum/glycerol hydrogel (a decrease of 0.3–0.4 eV in binding energy values) indicated the formation of hydrogen bonds between the oxygen of nanoclay Si–OH or Al–OH groups and hydrogen of the organic network, suggesting that both nanotube surfaces were modified [117]. Some particular cases require the combination of several different methods. For example, ^13^C nuclear magnetic resonance (NMR) spectroscopy and XPS data were combined [103] to establish the mechanism of polyphosphonium grafted HNTs synthesis. The XPS and NMR data, along with the FTIR findings, revealed the modification of halloysite via the redox-initiating system. Polyphosphonium grafted HNTs and alginate polysaccharide were then used for facile formation of an antibacterial hydrogel.

Besides spectroscopy, thermal analysis methods are also used for composition analysis. Evaluation of phase transitions as a function of temperature for biopolymer/HNTs composites can be done by DTA and TGA under N_2_ atmosphere. A quantitative analysis of TGA curves estimates the ratio between organic and inorganic parts in hybrid materials [92,101,103]. The mass of biopolymer in a sample is determined as the mass percent lost over the certain temperature range minus the mass loss of pristine halloysite. Organics degradation occurs in the 250–400 °C temperature range, and the corresponding mass loss over this range does not overlap with the decomposition of the hydroxyl groups of halloysite in the range of 480−550 °C. Quantifying the mass of protein adsorbed on halloysite by TGA provided greater accuracy compared to measuring the residual protein in solution after the adsorption process [93]. TGA can also be performed to compare the thermal degradation behavior of single biopolymer and different biopolymer/HNTs composites [96,97] as well as for evaluation of the drug loading efficiency [100].

All analytical techniques employed for analysis of biopolymer/HNT composites have their own strengths and weaknesses. Despite the great variety of analytical approaches (Table 1), most of them provide rather general information about a bulk sample. For instance, none of the aforementioned techniques allows obtaining reliable data on sample morphology (with high accuracy and resolution). Then, the HNT loading/grafting efficiency/quality remains rather doubtful, though the properties of the whole composite are highly dependent on the quality of HNT loading or grafting. Hence, the importance of comprehensive biopolymer/HNT composites investigation at the nano level is difficult to overestimate.

## 5. Electron Microscopy as the Ultimate Tool for Biopolymer/HNT Composites Analysis

Electron microscopy (EM) including scanning and transmission electron microscopies has become one of the most attractive cutting-edge analytical techniques thanks to the considerable progress in the development of electron guns and optics, detectors, signal processing and various installations for cryo/in situ/operando studies [118,145,146,147,148,149,150,151,152,153,154].

Nowadays, almost no credible research devoted to the study of complex biopolymer/HNT composite materials is complete without the use of EM data. Otherwise, the absence of this data raises immediately the question—whether the loading of compounds into the nanotubes was successful or not? The work [155] devoted to the synthesis of antimicrobial biodegradable composites based on pectin and HNTs loaded with rosemary essential oil is a good example illustrating the importance of EM data. Encapsulation of the essential oil (antimicrobial agent) inside HNTs with subsequent dispersion of as-obtained hybrid materials in pectin led to a much slower release of the rosmarinic acid molecules. However, the fact of rosemary oil encapsulation was not proved by any analytical technique, while the obtained dispersions of the primary hybrid materials (oil/HNT) were analysed only by TG-DTA and XRD. Both methods are a kind of “black box” since they do not allow judging about the homogeneity of hybrid material dispersion in the pectin matrix. Thus, despite the good antimicrobial activity of the obtained materials, enhanced mechanical properties and decelerated release of the rosemary essential oil, the absence of EM data makes the results rather unreliable.

Unlike other techniques used for the analysis of antimicrobial biopolymer/halloysite materials EM allows one to investigate a particular nanotube and, hence, to verify the antimicrobial composite at the nano level. This information is important because the effectiveness of the whole composite will strongly depend on the quality of loading/absorption into/onto HNTs. The scanning electron microscopy (SEM) and transmission electron microscopy (TEM) are rather different but complementary techniques. SEM is basically intended for the investigation of the sample surface/morphology. However, as a consequence of comparatively low accelerating voltage (≤30 kV) and, hence, not very high electron energy (≤30 keV), SEM resolution can vary between 0.4 nm and 20 nm. Unlike SEM, the electron beam of TEM has higher energy (≤200 keV, rarely ≤300 keV), better resolution (up to 0.5 Å in high-resolution mode (HRTEM)) and it penetrates through the sample. Thus, TEM is usually used for observation of the inner structure. Moreover, there are many types of electron-matter interactions (e.g., scattered/backscattered/diffracted electrons, characteristic X-rays, cathodoluminescence, auger electrons, etc.) that can be employed for the analysis of the samples in both types of electron microscope. In this regard, a comprehensive analysis of the analytical approaches to the study of antibacterial biopolymer/HNT composites using EM is of great importance and interest. It should also be underlined that the simple presence of EM data does not make research credible since many factors as sample preparation, observation conditions and most importantly, data interpretation can be done wrongly or insufficiently.

**Figure 3:**
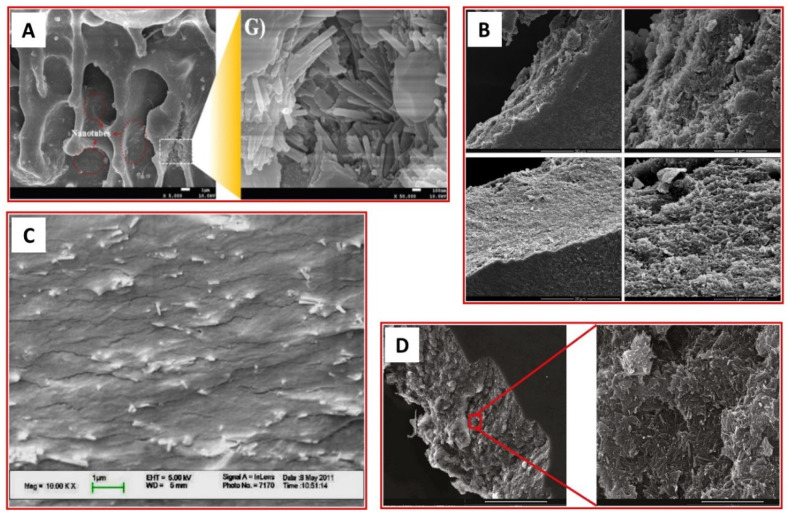
SEM images of the cross-section of various biopolymer/HNT films: (**A**) HNTs-EP combined with sodium alginate. Reproduced with permission from Ref. [103]. Copyright 2018 Royal Society of Chemistry, (**B**) HNTs combined with HM pectin (top) and LM pectin (bottom) Reprinted with permission from Ref. [127]. Copyright 2011 American Chemical Society, (**C**) HNTs combined with chitosan. Reproduced with permission from Ref. [121]. Copyright 2012 Elsevier, (**D**) HNTs combined with hydroxypropyl cellulose. Reprinted with permission from Ref. [125]. Copyright 2011 American Chemical Society.

SEM has been widely used for the investigation of the surface of bulk materials like antibacterial films, membranes and other hierarchical structures composed of halloysite and biopolymers [86,87,88,89,90,91,103,106,117,119,120,121,122,123,124,125,126,127,128,129,130,131,132,133]. All EM data including SEM images should provide unambiguous information about composite morphology and structure. Surprisingly, this obvious condition is not always satisfied. For instance, functional films with thermo-sensitive antioxidant/antimicrobial activity were obtained by incorporation into pectin matrix of HNTs modified by cucurbit [6] uril molecules and loaded with peppermint essential oil [91]. Despite the SEM studies of the modified HNTs, there were no cross-section views of the film, hence no information on halloysite distribution inside the film. The SEM photos of starch films reinforced with HNTs also lacked the cross-section photos even though some nanotubes were visible on the film surface [155]. In contrast to these two examples, the authors of [90,103,117,121,122,125,127] provided comprehensive information on halloysite distribution in polysaccharides with the help of cross-sectional SEM (Figure 3). It is worth mentioning that the preparation of an adequate cross-section for SEM investigation strongly depends on the sample and can be performed by cryofracturing [121,122] or ultramicrotomy [128]. The focused ion beam (FIB) is another way to investigate the bulk samples in depth by SEM, as was demonstrated in [119]. Apart from observation of the sample (polylactide film doped with lactic acid oligomer and HNTs) cross-section the 3D electron microscopy tomography was also employed revealing the nanocomposite dispersion in the film.

**Scheme 1:**
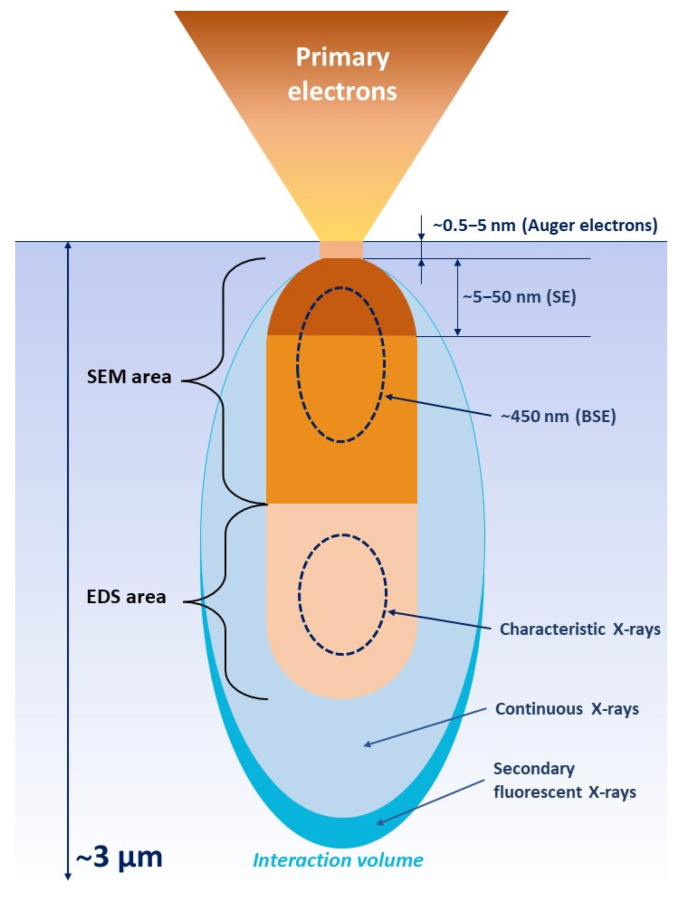
Electron–sample interaction in SEM.

As mentioned above, the electron–substance interaction has many forms and produces various kinds of signals simultaneously (Scheme 1). All of them convey different information about the sample. In SEM investigations two types of signal are usually detected: the backscattered electrons (BSE) and the secondary electrons (SE). The first type of electrons originates from a wide region within the interaction volume and occurs due to elastic collisions of electrons with atom’s electron shell, which causes a change in the trajectory of the electrons. Evidently, heavier atoms scatter stronger than light atoms and thus create a higher signal. The number of BSEs reaching the detector depends on the atom Z number and helps to distinguish between different phases (in other words, the phases containing the heavier elements are brighter than those with light elements). Furthermore, BSE images can also provide beneficial information on topography, crystallography and the magnetic field of the sample.

**Figure 4:**
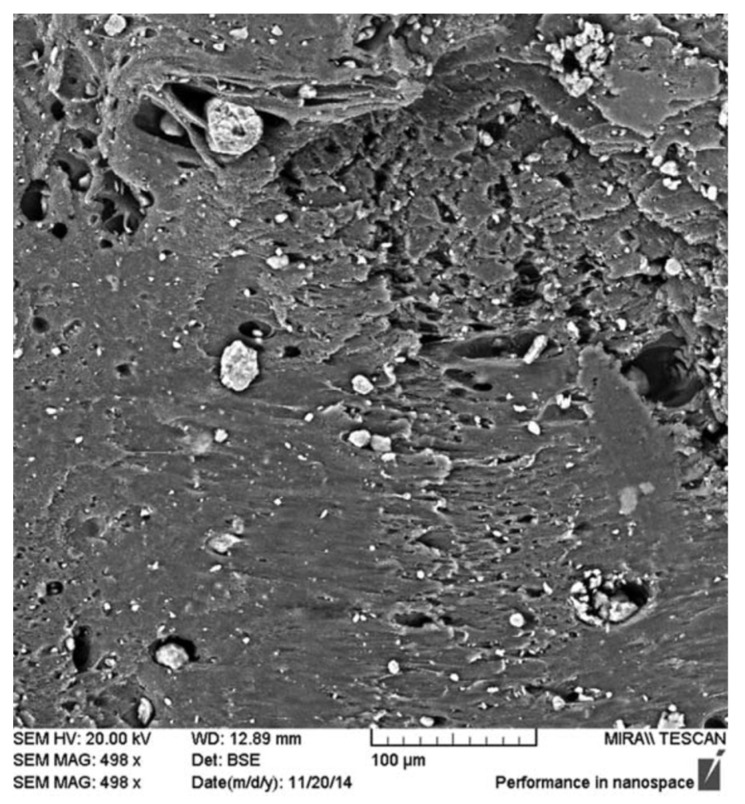
Backscattered electron (BSE)-SEM micrograph of the PLA/HNT composite. Reproduced with permission from Ref. [126]. Copyright 2015 John Wiley and Sons.

A good example of employment of BSE signal in SEM investigation was presented in the work [126], where HNTs were mixed with PLA in order to reinforce the latter. Since the elements constituting halloysite (Al, Si, O) are considerably heavier than those of PLA, the BSE micrograph clearly indicated the presence of higher density material as bright spots within the polymer matrix (Figure 4).

In contrast to BSE, SE originate from the surface or the near-surface regions of the sample. They occur due to inelastic interactions between the primary electron beam and the sample and contain lower energy than the backscattered electrons. SE are the best option for the investigation of the topography of the sample surface. The combination of both signal types could be beneficial owing to mixing of the contrasts of different natures.

**Figure 5:**
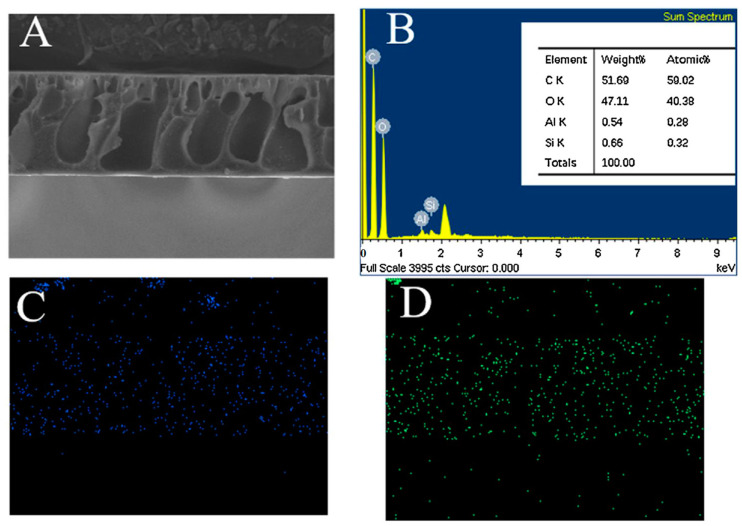
(**A**) SEM micrograph of the cross-section of 3 wt.% LPDHNTs/CA composite membrane, (**B**) the corresponding energy-dispersive X-ray spectroscopy (EDS) spectrum, (**C**,**D**) EDS mapping of Si and Al elements respectively. Reproduced with permission from Ref. [129]. Copyright 2017 Elsevier.

Besides BSE and SE, the primary electron beam generates X-rays (Scheme 1). The incident beam knocks out the electrons from an atom’s electron shell leaving a hole which will be eventually filled by electrons from the outer (higher energy) shell. Such an electron recombination is accompanied by emission of the characteristic X-rays. The number and energy of the X-rays emitted from a sample can be measured by an energy-dispersive spectrometer. As the energies of the X-rays depend on the energy difference between the two shells and on the atomic structure of the emitting element, energy-dispersive X-ray spectroscopy (EDX or EDS) allows studying the sample elemental composition. Taking into account that the electron beam is focused into the narrow probe, the characteristic X-rays can be received from discrete points of the sample surface and, hence, the elemental maps can be built. EDS is very powerful technique which can significantly improve SEM analysis [129,130]. For example, it was applied for investigation of cellulose acetate membrane doped with L-DOPA functionalized HNTs [129]. While the micrographs scale was large enough to see single nanotubes, their presence and distribution could be evaluated by EDX elemental mapping since halloysite was the only source of Al and Si atoms (Figure 5).

**Figure 6:**
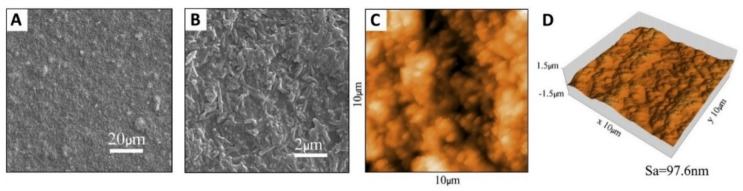
The examples of combination of (**A**,**B**) SEM and (**C**,**D**) atomic force microscopy (AFM) data to investigate halloysite nanotubes. Reproduced with permission from Ref. [124]. Copyright 2018 Elsevier.

Taking into account the great role of the surface roughness in biofilm formation, quantification of this parameter could be useful. Nonetheless, in the conventional set-up, SEM is not able to provide such information. In this case, the atomic force microscopy (AFM) or scanning force microscopy (SFM) providing sub-nanometer topography resolution can be combined with EM to comprehensively investigate the sample surface [90,124,129] (Figure 6).

Although SEM and AFM allow one to study rather large sample areas, these techniques provide information only from the sample surface (indeed the penetration depth of SEM electron beam strongly depends on sample nature and acceleration voltage and lies in the 1–5 µm range [132]). In order to observe what is loaded inside the halloysite lumen or/and adsorbed onto its outer surface, the TEM technique should be employed. However, TEM application for the analysis of biopolymer/HNT composites is associated with some problems.

**Figure 7:**
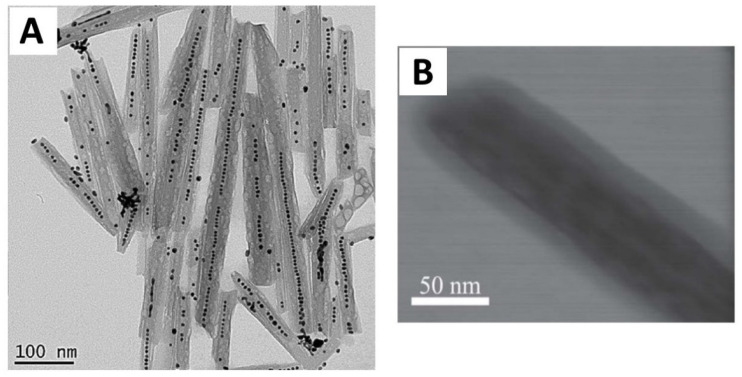
(**A**) an example of “bubbling” of HNTs under electron beam. Reprinted with permission from Ref. [132]. Copyright 2017 American Chemical Society, (**B**) example of TEM image shading due to organics degradation. Reproduced from Ref. [85] with permission from the Royal Society of Chemistry.

Firstly, the high energy electron beam of TEM (usually 200 keV) can considerably disrupt halloysite crystal structure (“bubbling” of HNT and disappearance of the discernible aluminosilicate nanosheets) and lead to the degradation of biopolymers (“burning” of the organic molecules and shading of the whole image) (Figure 7). These phenomena should not be confused with halloysite modifications (e.g., acidic etching of HNTs for lumen enlargement).

**Figure 8:**
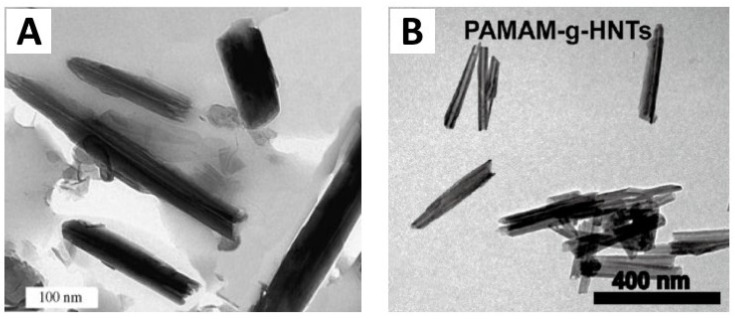
(**A**) HNTs after CaCO_3_ precipitation. Reproduced with permission from [133]. Copyright 2005 John Wiley and Sons, (**B**) polyamidoamine (PAMAM) grafted HNTs. Reprinted with permission from Ref. [102]. Copyright 2018 American Chemical Society.

Secondly, the TEM data can be wrongly or insufficiently interpreted. For instance, CaCO_3_ precipitation during urease-catalysed reaction inside HNT lumen was proved by XRD data and the absence of empty space inside nanotubes in TEM images (Figure 8A) [133]. Nevertheless, the conclusions drawn from EM data were rather doubtful, since some nanotube lumens were clearly visible in TEM, whereas the absence of a few can be explained by the thicker walls and the fact that nanotubes were immersed in viscous substance. The validity of TEM data, in this case, might be significantly enhanced by using selected-area electron diffraction (SAED) and conventional dark-field (DF) imaging available in every modern TEM. The latter would allow lighting up only vaterite phase inside the HNT lumen by choosing with the help of objective aperture the corresponding CaCO_3_ Bragg reflection. The analysis of the EM micrographs of polyamidoamine grafted halloysite nanotubes (PAMAM-g-HNTs) intended for siRNA delivery is another example of the confusing interpretation of the TEM data [102]. According to the authors’ claim a successful PAMAM grafting was illustrated by a “black layer of PAMAM polymer” located on the surfaces of HNTs (Figure 8B). Considering the case of the studies mentioned above [102,133], the greater contrast and, hence, the “blacker” HNTs in comparison with pristine halloysite cannot be a reliable sign of successful surface modification and might be explained by a CCD camera being beyond the dynamic range. Summing up, special attention should be paid to the TEM data interpretation.

**Figure 9:**
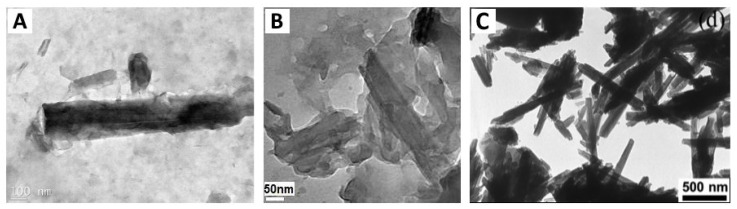
(**A**) HNTs wrapped by DNA. Reproduced with permission from Ref. [86]. Copyright 2008 IOP Publishing, Ltd., (**B**) HNTs modified by lysozyme. Reproduced with permission from Ref. [134]. Copyright 2015 Royal Society of Chemistry, (**C**) HNTs grafted by chitosan. Reproduced with permission from Ref. [99]. Copyright 2016 Royal Society of Chemistry.

Lastly, unlike antimicrobial HNT composites with inorganic compounds for which the presence of, for example, Au nanoparticles inside the HNT lumen is obvious and well visible in TEM images (Figure 7A), the organic/bio-molecules in biopolymer/HNT composites are sometimes hardly detectable. For example, in some cases, the TEM images could not provide doubtless information about halloysite outer surface modification by sodium salt of double-stranded DNA [86] or lysozyme [134], since HNTs were immersed in irregular viscous precipitations (Figure 9). The presence of DNA on the outer surface of HNTs can be additionally confirmed by a change in the nanotube surface adhesion measured by AFM [135].

Sometimes in order to confirm HNT modification by different biopolymers, the lumen and external diameter of modified HNTs could be measured and compared with those of pristine halloysite [99,100,101]. To reduce errors the use of monodisperse nanotubes (HNTs with similar outer diameters) and choosing the appropriate TEM magnification are highly recommended. So, the disregard of these requirements at the measurements of the outer diameters of raw and chitosan grafted HNTs (Figure 9C) led to huge error bars: the average diameter of raw halloysite was 43.5 ± 18.5 nm, while that of the modified one was 74.1 ± 22.3 nm [99]. The diameter difference of 30 nm is hardly seen at a scale of 500 nm resulting in a greater error.

**Figure 10:**
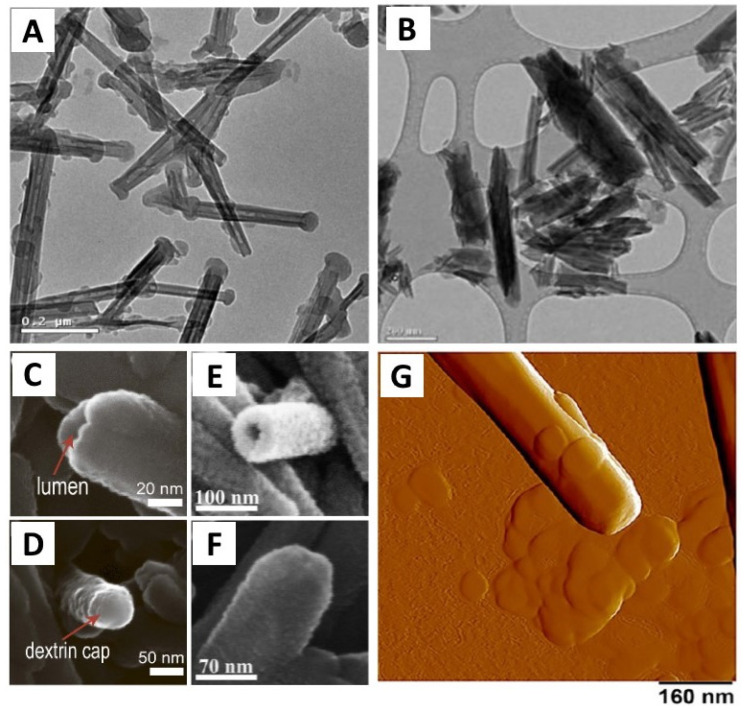
(**A**) TEM image of HNTs with end stoppers of starch complex. Reproduced with permission from Ref. [81]. Copyright 2013 Elsevier; (**B**) TEM image of HNTs loaded with thyme oil and capped with sodium alginate. Reproduced with permission from Ref. [136] Copyright 2017 John Wiley and Sons; (**C**) SEM images of empty HNT and (**D**) HNTs loaded with brilliant green and protected with a dextrin caps. Reproduced from Ref. [137] Copyright 2015 Dzamukova, Naumenko, Lvov, Fakhrullin; (**E**) SEM images of empty HNTs, (**F**,**G**) SEM and AFM images of HNTs loaded with curcumin and capped by dextrin caps. Reprinted with permission from Ref. [75]. Copyright 2019 American Chemical Society.

The capping of HNTs with biopolymeric end-stoppers is a very popular approach to slow down release kinetics (to days and weeks) and, thus, to obtain long-lasting effects of the antimicrobial agents loaded in the lumen. However, visualization and observation of HNT with end stoppers is not always obvious as presented in Figure 10A. Thus, rather questionable results of the TEM investigations were provided after encapsulation of thyme essential oil and capping of HNTs with sodium alginate in the work [136], since halloysite end stoppers were not clearly visible in the presented micrographs (Figure 10B). SEM and AFM can help to overcome this problem as was shown elsewhere [75,137] (Figure 10C–G). Therefore, it is always necessary to keep in mind the complementarity of the different types of microscopy.

**Figure 11:**
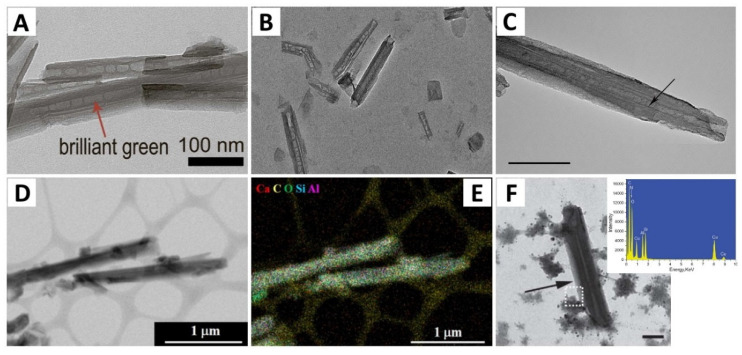
TEM images of HNTs loaded with (**A**) brilliant green. Reproduced from Ref. [137] Copyright 2015 Dzamukova, Naumenko, Lvov, Fakhrullin, (**B**) norfloxacin. Reproduced with permission from Ref. [106]. Copyright 2020 Elsevier; (**C**) binase. Reproduced from Ref. [138]. Copyright 2017 Khodzhaeva, Makeeva, Ulyanova, Zelenikhin, Evtugyn, Hardt, Rozhina, Lvov, Fakhrullin and Ilinskaya; (**D**) TEM image and (**E**) EDX elemental mapping of HNTs loaded with calcium alginate Reprinted with permission from Ref. [139]. Copyright 2018 American Chemical Society; (**F**) TEM image of chitosan grafted HNTs and the corresponding EDX spectrum from highlighted zone. Reproduced with permission from Ref. [140]. Copyright 2013 Royal Society of Chemistry.

The detection of biopolymer loading inside HNT is rather challenging. Sometimes the loaded biopolymer has sufficient Z contrast to be visible in TEM (Figure 11A–C). Nevertheless, in some cases, TEM cannot provide obvious visual confirmation of successful loading. For example, the calcium alginate hydrogel formation confined by the HNT lumen was difficult to verify by TEM alone [139]. In this regard the authors employed the EDX technique to register Ca atoms, and, therefore the presence of calcium alginate inside the halloysite (Figure 11D,E). Another example of successful EDX application for the analysis of chitosan-grafted HNTs was presented in work [140]. The chitosan layer has too low Z contrast to be seen in TEM of medium magnification (Figure 11F). However, the EDX analysis of the HNT border (highlighted zone in Figure 11F) revealed the presence of nitrogen, a constituent of chitosan molecules. Thus, the authors were able to prove their hypothesis of successful chitosan grafting to HNT outer surface, using EDX technique as a support to rather doubtful TEM image. Surprisingly, even though energy-dispersive X-ray spectroscopy and elemental mapping available in scanning transmission mode (STEM) are reliable and powerful tools for the discrimination of the substances of different chemical compositions, their utilization for the analysis of biopolymer/HNTs antimicrobial composites is comparatively rare.

Finally, given the low visibility of biopolymers in TEM, positive and negative staining of biomolecules could be the solution. The term “staining” implicates applying a heavy metal salt stain either to a sample for observation or to its environment. Indeed, the staining technique has been applied for TEM studies of various biological objects (e.g., bacteriophages, viruses, bacteria, antibodies) and biomolecules (e.g., nucleic acids, proteins) [156,157,158,159]. Although biopolymers and their composites have been investigated by TEM involving a staining technique (see [141,142,143,144]) the application of staining in TEM studies of biopolymer/HNT composites is very rare. One of the exceptions is the work [139], in which AgNO_3_ was added to HNTs loaded with calcium alginate as a staining solution. Ag^+^ is promptly reduced by alginate and the formation of Ag nanoparticles is expected in the proximity of the polysaccharide.

**Scheme 2:**
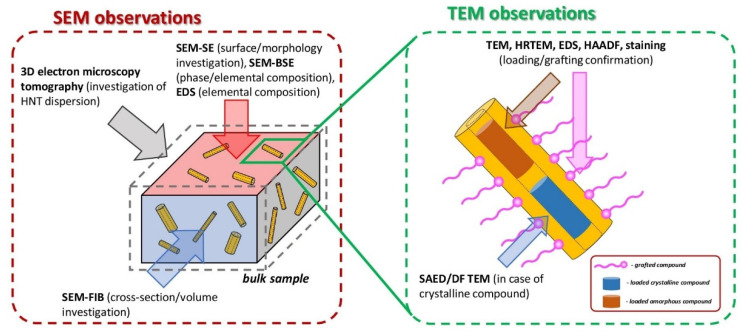
Complementarity of SEM and TEM techniques for characterization of HNT/biopolymer composites.

Summing up, employment of a suitable strategy for the investigation of biopolymer/HNTs composites by EM is of the great importance. Many parameters such as observation conditions, sample preparation and the choice of the appropriate analytical approach providing reliable data have a dramatic impact on the final result. Special attention should be drawn to the combination of different analytical techniques available in modern electron microscopes. The Scheme 2 summarizes these analytical techniques as well as the ways of their application.

## 6. Advantages and Limitations of Electron Microscopy

Thanks to superior resolution of modern electron microscopes, EM is probably the best form of comprehensive investigation of the morphology and structure of halloysite-based antimicrobial composites at the nano level. As various types of electron-matter interactions happen when electron beam hits the sample additional information (e.g., chemical composition, crystal structure) can be retrieved from the same area with the help of specialized detectors, cameras and software developed for EM and making it a universal analytical technique.

However, there are still some limitations of EM in the investigation of antimicrobial materials containing halloysite and biopolymers. The EM is a very local technique. This natural limitation cannot be overcome, hence microscopy data cannot be extrapolated to the entire sample and must be supported by more “general” analytical methods like IR/Raman spectroscopy, XRD, XRF, TGA, etc.

It should be underlined that EM is, first of all, a visualization tool. A routine EM study does not provide quantitative information on polymer contents in halloysite/biopolymer composites and amounts of the encapsulated antimicrobial agents can be evaluated only theoretically (usually on the basis of the concentration of the agent solution [160]). The spectroscopic methods (e.g., EDS) available in electron microscopes can provide semi-quantitative information if a number of criteria are considered. For example, the reliability of EDS data can depend on the sample morphology and matrix, along with microscope settings. The sample must be flat, otherwise, the X-rays can be completely blocked. In other cases, the sample matrix may absorb low energy X-rays more than higher energy X-rays, which can create errors in the quantitative results. The position of the sample in relation to the EDS detector position is important in topographic studies. Lastly, the accelerating voltage must be sufficient (usually 1.5–2 times higher than the energy of the X-ray lines of interest) for effective excitation of the elements found in the sample. There should also be an adequate probe current to produce an X-ray count rate that is statistically significant. Summing up, the limits of detection of the modern EDS systems are ≥0.1% for heavier elements and ≥1% for lighter elements (F to Be) [161]. Unlike EDS, wavelength-dispersive X-ray spectroscopy (WDS) might be considered an option to obtain better energy resolution at higher count rates (in other words, the amount of overlap between peaks of similar energies is much smaller in the WDS system). Nevertheless, being significantly more expensive than EDS, WDS has its own disadvantages (slow collection times, only small areas detection is possible) and it is still a semi-quantitative technique [162].

The lower probability of electron interaction with light elements (or low Z elements) causes bad visibility of biopolymers on the HNT surface or inside the nanotube lumen. Fortunately, this problem can usually be overcome. For instance, the high-resolution mode could be used in some cases, yet this may be dangerous for the sample. The decrease of accelerating voltage (e.g., from 200 to 120 kV) can considerably improve visibility of “light” materials. Electrons with lower kinetic energy (e.g., 120 keV) will scatter more on the specimen, thus, giving the better contrast. Nevertheless, the rising chance of inelastic interactions of “slow” electrons with the specimen and, hence, high probability of its degradation under the beam should be considered. Finally, the employment of the already existing staining protocols and the development of new ones could be another solution to this issue.

The sample preparation for EM observations is also of the great importance and strongly affects the quality of the information obtained. Since the deep vacuum must be maintained in the microscope column, the sample has to be dehydrated (in particular it concerns the biological objects like cells, macromolecules, proteins). In order to keep the shape and morphology of the sample during dehydration a freeze-drying method is usually applied [163,164,165,166]. However, despite the freeze-drying finding widespread application in industry and academic fields, it still has some drawbacks that could create image artifacts [163]. Thus, the growing ice crystals can damage cell ultra-structure by exerting pressure in the adjacent cytoplasm, membranes, and structural proteins. Furthermore, the freezing out water changes pH in the unfrozen cytoplasm which results in the abnormal cross-linking of macromolecules, and the denaturation and precipitation of proteins.

Placing of the conducting film (e.g., carbon, platinum, gold or gold alloys) on the sample surface is another requirement in a SEM investigation, unless a sample is conductor. The goal is to avoid the accumulation of electrical charges on the sample surface leading to a repulsion of the primary beam, and impeding obtaining a useful image. Metallization also helps to avoid damage to the sample due to the heating caused by the electron beam [161]. However, the application of metallization is sometimes undesirable since it can considerably modify important sample surface information such as fine roughness or porosity. The lines of elements of the deposited film are also detected by EDS and can overlap with those of interest leading to the loss of important sample information.

Environmental electron microscopy or low vacuum electron microscopy could be an alternative solution for the observation of non-conductors and/or hydrated samples [127,160,161,167,168,169,170,171]. The samples surrounded by water vapour or inert gas can be observed without any preparation thanks to a special construction of environmental electron microscopes and their pumping systems. The gas or water molecules present in the specimen chamber are ionized by a primary electron beam. The positively charged ionized molecules are attracted by the negative charges accumulated on the sample surface restoring the electrical and temperature equilibrium on it. Meanwhile electrons removed from gas atoms by ionization are collected by the detector and the corresponding image is formed providing topographical information similar to that obtained with SE [172]. The environmental scanning electron microscopy (ESEM) was effectively applied to the different biopolymeric capsules (garlic extract entrapped by fat; salt particles coated by gelatine or fat; a wet alginate bead) providing data on the quality of capsule fabrication process and the functionality of the capsule original surface [173]. It should be noted that our literature review did not reveal any attempt to use environmental EM for the study of HNTs/biopolymer composites.

In situ electron microscopy is an alternative and even more powerful approach to the study of samples containing water or requiring special handling. The key idea of in situ EM is placing the sample in hermetically sealed cells/chips with transparent windows and its study at “ambient” conditions. As it follows from Table 2 presenting the in situ TEM solutions from Protochips and DENSsolutions the scope of the conditions for in situ TEM observations is very wide. Thus, employing the liquid or gas TEM holders allows the detailed investigation of wet samples or even the samples in liquid (e.g., water or buffer solution with a given pH) and gas media [174,175,176,177,178,179,180]. For example, in situ TEM could be used to image bacteria and the process of their tellurite reduction with no significant damage neither from the sealing nor from the electron beam [181]. Besides static observations, various processes can be observed and studied as was done for the interaction of brain tumour stem cells with nanoparticles [182]. The data obtained can help the understanding and improvement of the nanoparticle-based therapy. In situ EM might be used not only for the observation of biopolymer/HNT composites in favourable conditions but also for studying the antimicrobial activity of the composites at the nano level by monitoring the drug release process.

The sample tolerance towards the electron beam is the last EM limitation but not the least important one. As it was mentioned above, HNT and a majority of biopolymers (and biological samples in general) are prone to degradation under continuous TEM radiation (e.g., Figure 7). In spite of the fact that SEM radiation is less energetic than TEM one, substances like fat with low fusion temperature are still very sensitive and require a decrease of either vacuum level or electron dose [173]. The latter is also of a great importance in in situ EM since high energetic electrons induce water radiolysis which might be harmful due to oxygen and hydroxyl radicals [177,180].

A few strategies to overcome the problem of sensitive samples might be suggested. The most straightforward way to reduce the electron dose is to set the shorter acquisition time (in case of SEM and STEM, a shorter dwell time, i.e., increasing scanning speed). The choice of the smaller condenser aperture and spot size (from smaller to larger values) might also be beneficial. Since, these operations significantly limit spatial/temporal resolution and the study length, hence, the specially designed ultrafast sensitive CDD and CMOS cameras are required. Implementation of the cryogenic temperatures to sensitive specimens and their investigation at higher electron doses is an alternative. The successful investigation of many biological samples proves the effectiveness of cryo-EM [146,167,183,184,185]. The cryogenic temperatures can be obtained with the help of TEM cryo-holders and SEM cryo-chambers supplied by many manufactures (e.g., GATAN, JEOL, FEI, etc.). Cryo-electron microscopy is also effective in imaging of wet samples without causing drying artifacts, which may be very useful for biopolymer composite materials.

Summing up, the possibility to provide sample investigation under different conditions makes EM a versatile analytical technique. As with every analytical technique, it has limitations. Nevertheless, as has been demonstrated above, the majority of them can be overcome thanks to recent developments of the EM hardware, new analytical approaches and signal processing algorithms making EM an (almost) ultimate tool for investigation of biopolymer nano-composites.

## 7. Conclusions

Biofilms and their harmful effects on our everyday life are one of the contemporary challenges facing researches all over the world. As a result of the battle against biofilms, that began in the last century and is still underway, a number of antibiofilm solutions have emerged. Due to a great environmental concern, “green strategies” involving the use of various biopolymers attracted considerable attention of research. The biopolymer composite materials revealed even higher antimicrobial activity owing to their better penetration through the biofilm matrix and thus enhanced antibiotics delivery. Additionally, the composites can provide firm primary fixation of an antimicrobial agent on the surface and controlled biocide release for a certain time period. In this regard, a special interest was drawn to the halloysite nanotubes, since they are natural aluminosilicate nanorolls possessing specific physical properties, outstanding mechanical stability, high biocompatibility and low price. A number of studies have been devoted to the synthesis of biopolymer/halloysite antimicrobial composites and to the investigation of their properties. Adequate analysis of such materials is of high importance, since their overall antibiofilm activity strongly depends on the quality of nanotube loading/grafting with antimicrobial agents. Electron microscopy, a powerful and versatile analytical technique, which allows obtaining detailed information about the composites at the nano level seems to be the best tool.

In this paper, the recent contributions of electron microscopy to the studies of halloysite/biopolymer composites were reviewed. Despite the fact that electron microscopy proposes a wide variety of different approaches to the analysis of halloysite based antimicrobial materials, its application in this field has usually been limited by image acquisition. In this regard, a few suggestions on the future development/implementation of this analytical technique to the halloysite-based antimicrobial composites as well as highlighting the main challenges can be made.

Most importantly, there should be careful choice of the observation conditions and adequate data interpretation. For example, an appropriate electron dose is required to avoid halloysite and biopolymer degradation. More frequent use of environmental and cryo-microscopy to enhance sample tolerance towards electron beams can also be considered. The electron microscopy micrographs should be corroborated by complementary techniques like energy-dispersive X-ray spectroscopy. The correct visualization of drug encapsulation in nanotubes is a clue to a proper evaluation of the loading quality and, therefore, to the prediction of the effectiveness of the whole antimicrobial composite. However, it is still rather difficult, because of the low Z-contrast of biopolymers. Thus, the use of staining techniques and the development of new staining protocols are of great importance. In situ TEM, one of the most recently developed methods is another great opportunity to study antimicrobial composites and their activity. Finally, taking into account that electron microscopy is still used only to visualize the encapsulated material inside halloysite nanocontainers, the development of new TEM protocols providing semi-quantitative/quantitative data is of great importance and seems to be a major challenge in coming decades.

## Figures and Tables

**Table 1:** Comparison of analytical techniques employed for biopolymer/halloysite nanotube (HNT) composite analysis (“+” means that the method is suitable for determination of a given characteristic of biopolymer/HNT composite, “±” means that the method is of limited suitability and “–” means that the method is unsuitable).

Analytical Technique	Ref.	Information Obtained					
		Chemical Structure/ Composition	Morphology/ Bulk Information	Crystal Structure	Particles Size	HNT Surface Modification Confirmation	HNT Loading Efficiency
FTIR	[92,96,97,99,100,101,102,106,117]	+ (the finger-print bands of the corresponding functional groups)	–	–	–	+ (appearance of new bands relating to chemical bonds/phase formation)	–
XRD	[96,98,100,102,106,118]	–	–	+	–	+ (at condition new phase is crystalline)	–
TGA	[92,93,96,97,100,101,103]	+ (by loss of organic content)	–	–	–	+	+ (high accuracy)
XPS	[102,103,117]	+	–	–	–	+	–
DLS	[102,111]	–	–	–	+ (dispersions of spherical particles 0.1 nm–10 μm)	± (small change in size)	–
Zeta potential	[100,102]	–	–	–	–	+ (significant change)	+ (rough estimation by distribution)
NMR	[103]	+	–	–	–	± (not universal method)	± (not universal method)
UV-Vis- near IR spectroscopy	[97,100,116]	–	–	–	–	± (not universal method)	+ (in solution after antimicrobial agent release)
Optical microscopy	[108,109,110]	–	+ (relatively low resolution)	–	+ (particles > 1 μm)	–	–
Confocal microscopy	[82,93,101]	–	+ (fluorescent tag or staining are necessary)	–	+ (particles > 0.5 μm)	± (not universal method)	± (not universal method)
SEM	[86,87,88,89,90,91,96,103,106,117,119,120,121,122,123,124,125,126,127,128,129,130,131,132,133]	+ (EDS or WDS)	+ (FIB, electron tomography for bulk information)	+ (EBSD)	+	+	–
TEM	[81,86,99,100,101,102,106,131,133,134,135,136,137,138,139,140,141,142,143,144]	+ (EDS)	+	+ (SAED, HRTEM)	+	+	+

**Table 2:** Comparison of in situ TEM solutions from DESsolutions and Protochips.

DENS Solutions			Protochips		
Wildfire (in situ TEM heating)	Heating control	4-point probe heating	Fusion Select (in situ TEM heating & biasing)	Temperature Range	RT to 1200 °C (900 °C for Electrothermal)
	Temperature range	RT to 1300 °C			
	Temperature accuracy	≥95%		Temperature Accuracy	95%
	Temperature Homogeneity	≥99.5%		Temperature Stability	<0.01 °C
	Viewable area	850 µm^2^		Maximum Voltage	55 V (inquire for higher voltages)
Ligthning (in situ TEM biasing & heating)	Heating & biasing control	Closed 4-point probe feedback loop			
	Temperature range	RT to 1300 °C		Heating and Cooling Rate	programmable, any rate up to 1000 °C/ms
	Membrane breakdown voltage	≥150 V at RT/900 °C			
	Attainable E-fields	≥300 kV/cm at RT/900 °C		Current Range	Electrical Standard Resolution (ESR) 100 pA to 100 mA
	Detectable current range	1 pA to 100 mA			
	AC measurement	Up to 100 Hz			
	Temperature accuracy	≥95%			Electrical High Resolution (EHR) 2 pA to 100 mA
	Temperature homogeneity	≥99.5%			
Stream (in situ TEM liquid/biasing or heating)	Liquid thickness	<500 nm	Poseidon Select (in situ electrochemistry/liquid heating	Configurable E-chip Spacer	microwells, 0 nm, 50 nm, 150 nm, 500 nm, 1 μm or 5 μm
	Resolution	≤3 Å		Resolution	2 ± 1 nm or better
	Liquid pressure range (accuracy)	200–4000 mbar (±2 mbar)		Flow Ports	3 (static, flow or mixing)
	Liquid modes	Static, flow (infusion, withdrawal)			
	Liquid flow range	0 to 8 µL/min			
	Flow control: Direct closed loop feedback control	<10 s		E-chip Sealing Method	1 gasket
	Temperature range	RT to >100 °C			
	Temperature stability	±0.01 °C		Heating	RT to 100 °C closed-loop control
	Voltage range	−10 V to +10 V		Vapor Introduction	Software-controlled introduction of water,
	Current range	From pA to mA			
	AC impedance frequency range	10 uHz–1 M Hz			
Climate (in situ TEM gas & heating)	Resolution	≤100 pm	Atmosphere (in situ TEM gas & heating)	Resolution	<1.5 Å
	Gas mixing method	Continuous		Gas Mixing	0.01–99.99% mixtures of up to 3 gases via volumetric blending methanol, ethanol, hexane, naphtha, etc.
	Gas switching	≤15 s			
	Gas input lines	3			
	Gas flow range (normalised)	0–1 mL/min			
				Flow Rate	0.005–1.000 mL/min
	Pressure range	0–2000 mbar		Holder Base Pressure	3.0 × 10^−2^ Torr
	Heating range	RT–1000 °C		Operating pressure range	1.0–760.0 Torr
	Temperature stability	≤±0.01 °C		Temperature	25–1000 °C
	Heating control mode	4-point probe resistive feedback			
	Heating & cooling rate	Up to 150 °C/s			

## Data Availability

Not applicable.
